# Annual Renal Ultrasound May Prevent Acute Presentation With Acetazolamide-Associated Urolithiasis

**DOI:** 10.1212/CPJ.0000000000000761

**Published:** 2021-02

**Authors:** Karen J. Suetterlin, Vinojini Vivekanandam, Natalie James, Richa Sud, Sarah Holmes, Doreen Fialho, Michael G. Hanna, Emma L. Matthews

**Affiliations:** MRC Centre for Neuromuscular Diseases (KJS, VV, NJ, SH, DF, MGH, ELM), Queen Square Institute of Neurology, UCL and National Hospital for Neurology and Neurosurgery; and Neurogenetics Unit (RS), National Hospital for Neurology and Neurosurgery, Queen Square, London, UK.

**PRACTICAL IMPLICATIONS**

Annual renal ultrasound may prevent acute presentation with ureteric obstruction from acetazolamide-induced renal calculi.

Acetazolamide is used as first-line treatment for many neurologic channelopathies and as add-on therapy for other conditions such as epilepsy, idiopathic intracranial hypertension, and migraine. There is no randomized controlled trial evidence of its efficacy in genetically confirmed muscle channelopathies.^1,2^

Most adverse events associated with acetazolamide therapy are considered benign or tolerable, but fatal agranulocytosis, aplastic anemia, and an increased incidence of renal calculi have been reported.^3,4^ Despite this, routine ultrasound monitoring for the development of renal calculi is often not performed.

We reviewed records of all patients prescribed acetazolamide by our national channelopathy service between 2011 and 2018 as part of a service evaluation approved by our hospital audit committee. Patients with a genetically confirmed channelopathy who had been prescribed acetazolamide and had at least 1 posttreatment clinic appointment were included (minimum follow-up period of 6 months). Acetazolamide efficacy was determined by patient report of reduced frequency, duration, or severity of symptoms. Adverse effects were clinician, investigation, or patient-reported events that occurred while taking acetazolamide and not clearly attributable to an alternative cause.

Fifty-eight patients with genetically confirmed channelopathy were included. All but one of these took the immediate release formulation. The self-reported efficacy rate for acetazolamide varied with genetic diagnosis (figure, A).

**Figure:**
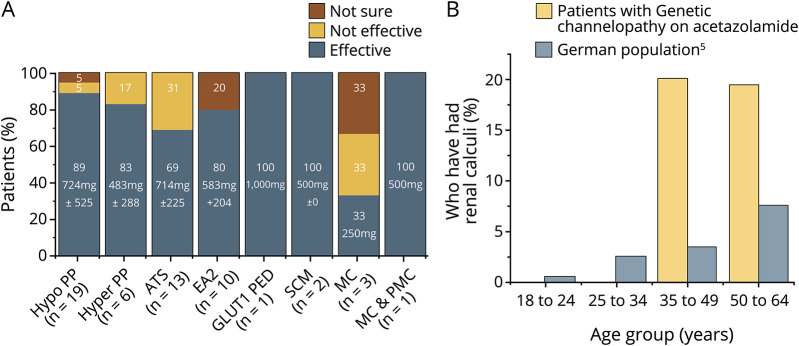
Safety and Efficacy of Acetazolamide in Genetic Channelopathies (A) Patient-reported acetazolamide efficacy by genetic diagnosis. Mean effective dose of acetazolamide and number of patients with efficacy data are also given for each genetic diagnosis. (B) Prevalence of renal stones in patients with genetic channelopathy taking acetazolamide compared with published data looking at the population prevalence of renal stones in a comparable European country (Germany in 2001). In the German study, a representative sample of the German population (7,500 people) was questioned on the incidence of renal stones during their life time. As in our cohort of patients with channelopathy, some of the renal stones reported in this group had been asymptomatic and identified as a result of medical imaging. The incidence of renal stones found in our patients with genetic channelopathy on acetazolamide is given in brackets for each age group. ATS = Andersen-Tawil syndrome, EA2 = episodic ataxia type 2, GLUT1 PED = paroxysmal exertion-induced dyskinesia, SCM = sodium channel myotonia, MC = myotonia congenita, PMC = paramyotonia congenita; Hyper PP = hyperkalaemic periodic paralysis; Hypo PP = hypokalaemic periodic paralysis.

Paresthesia was the most frequent side effect (24%) but was generally well tolerated unless it affected the face. Paresthesia occurred at doses as low as 125 mg. No blood dyscrasias occurred. Seven patients (12%) developed renal calculi while taking acetazolamide. There was no correlation between dose or duration of acetazolamide therapy and urolithiasis (*p* = 0.6 and *p* = 0.8, respectively, Student *t* test with Welch correction).

Six of the 7 renal calculi were detected on routine ultrasound monitoring. Four of these 6 eventually required elective lithotripsy, 1 required monitoring without intervention, and for 1, it was not known. The seventh patient required emergency lithotripsy when she presented to the emergency department in extremis with acute ureteric obstruction. This patient had not had the recommended yearly renal ultrasounds.

Seventy percent of patients with acetazolamide-associated renal calculi chose to continue acetazolamide under urology supervision, as it was the only effective treatment for their channelopathy. This was despite 60% of them developing further calculi and requiring repeat lithotripsy.

## Discussion

The prevalence of renal calculi was increased between 4- and 7-fold in patients with channelopathy older than 35 years treated with acetazolamide compared with the general population (figure, B).^5^ This was irrespective of the dose or duration of treatment.

As the vast majority (90%) of monitoring ultrasounds were performed locally and results communicated to us, we cannot be certain that every patient had the recommended annual ultrasound. Nevertheless, patients who did had their renal stones diagnosed before ureteric obstruction could occur allowing for elective lithotripsy when needed. In contrast, 1 patient who did not have the recommended monitoring presented acutely with complications requiring emergency intervention.

Note was also made of 1 patient receiving acetazolamide but not included in the selection criteria (genetically unconfirmed). This patient also presented with urolithiasis causing ureteric obstruction and acute renal failure after failing to receive monitoring scans. The severity of their presentation was increased by previously unidentified renal agenesis. Renal agenesis in the general population is common, 1 in 1,000.^6^ This suggests that a baseline scan before commencing acetazolamide is warranted, as acetazolamide's complications may be further exacerbated in those with congenital abnormalities.

This study demonstrates that acetazolamide can be effective for Andersen-Tawil syndrome. The patient-reported efficacy rate for episodic ataxia type 2 (80%) and hyperkalemic periodic paralysis (83%) is in keeping with that published previously.^1,2^ The efficacy rate for hyperkalemic periodic paralysis (89%) was higher than previous reports.^1,7^ This is potentially because all patients had arginine to histidine genetic mutations predicted to be the most acetazolamide responsive.^7^

Although this is a retrospective study and comes with the associated limitations, in the absence of randomized controlled trial data, our clinical experience of efficacy, monitoring, and safety data provide a useful resource to help counsel patients before treatment of genetic channelopathies. In addition, our monitoring and safety findings have important implications for any neurologist prescribing acetazolamide, e.g., for epilepsy, migraine, or idiopathic intracranial hypertension, as treatment-associated renal stones are not specific to channelopathies.^3,4^

In summary, our study suggests that regular ultrasound monitoring may reduce acute presentation with ureteric obstruction from acetazolamide-induced urolithiasis. A prospective study with a control group is required to confirm this. However, in the absence of such a study, we suggest that a baseline scan be performed before commencing acetazolamide therapy and annual ultrasound monitoring continues for the duration of treatment.
